# Potential injectable hydrogels as biomaterials for central nervous system injury: A narrative review

**DOI:** 10.1002/ibra.12137

**Published:** 2023-11-30

**Authors:** Santa Sarma, Dhruva J. Deka, Prakash Rajak, Damiki Laloo, Trishna Das, Purbajit Chetia, Dipankar Saha, Alakesh Bharali, Bhargab Deka

**Affiliations:** ^1^ Girijananda Chowdhury Institute of Pharmaceutical Science Assam Science and Technology University Guwahati Assam India; ^2^ Department of Pharmaceutical Sciences Dibrugarh University Dibrugarh Assam India; ^3^ School of Pharmaceutical Sciences Girijananda Chowdhury University Guwahati Assam India; ^4^ Department of Pharmacology NETES Institute of Pharmaceutical Science, Nemcare Group of Institutes, Mirza Guwahati Assam India

**Keywords:** biomaterials, brain repair, cell therapy, hydrogels

## Abstract

Numerous modalities exist through which the central nervous system (CNS) may sustain injury or impairment, encompassing traumatic incidents, stroke occurrences, and neurodegenerative diseases such as Alzheimer's disease and Parkinson's disease. Presently available pharmacological and therapeutic interventions are incapable of restoring or regenerating damaged CNS tissue, leading to substantial unmet clinical needs among patients with CNS ailments or injuries. To address and facilitate the recovery of the impaired CNS, cell‐based repair strategies encompass multiple mechanisms, such as neuronal replacement, therapeutic factor secretion, and the promotion of host brain plasticity. Despite the progression of cell‐based CNS reparation as a therapeutic strategy throughout the years, substantial barriers have impeded its widespread implementation in clinical settings. The integration of cell technologies with advancements in regenerative medicine utilizing biomaterials and tissue engineering has recently facilitated the surmounting of several of these impediments. This comprehensive review presents an overview of distinct CNS conditions necessitating cell reparation, in addition to exploring potential biomaterial methodologies that enhance the efficacy of treating brain injuries.

## Graphical abstract

Injectable biomaterials, such as hydrogels, combined with stem cells, offer promising strategies for repairing central nervous system (CNS) injuries. These biomaterials provide structural support and create a favorable microenvironment for cell survival and differentiation. Stem cells possess the potential to differentiate into specialized neural cells, promoting tissue regeneration and functional recovery. This innovative approach holds great potential for enhancing CNS repair and restoring neurological function.

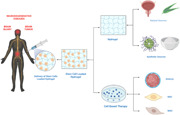

## 1 INTRODUCTION

The brain, being an intricate part of the central nervous system (CNS), is responsible for integrating and regulating information in the nervous system along with the spinal cord. Injuries to the CNS caused by diseases, disorders, or accidents are very critical due to the limited regenerative capacity of CNS tissues, intricate neural circuitry, and complex inflammatory responses. The presence of the blood–brain barrier (BBB) restricts the entry of therapeutic agents into the CNS, and the formation of glial scars at the lesion can hinder axonal regrowth. Secondary injury processes, including excitotoxicity and oxidative stress, further exacerbate damage. Timing of therapeutic interventions, heterogeneity of injuries, ethical concerns, and patient‐specific variability add to the complexity. Moreover, stroke, trauma, and a variety of neurodegenerative disorders can cause damage and death of neuronal tissue in the CNS. In view of the limited regenerative capacity of the CNS, damage to the CNS tissue can have severe consequences on the individual. Since CNS injuries have a profound impact on our society and existing pharmacological treatments are not effective enough, novel therapeutic approaches that target the regeneration of CNS tissue are essential.^1^

To address and facilitate the recovery of the impaired CNS, cell‐based repair has emerged as a mechanism for replacing damaged or degenerating cells and repairing CNS tissues. The therapeutic potential of cell‐based therapies lies in the reconstruction of damaged circuitry via (a) replacement of damaged neurons directly, (b) secretion of therapeutic factors enhancing neuroprotection, anti‐inflammatory effects, or enhanced angiogenesis in the pathological host environment, and (c) creating an environment that promotes neuroplasticity in the host. This cell‐based repair strategy encompasses the utilization of cellular entities to substitute impaired or depleted brain cells. This method entails employing either autologous cells, derived from the patient's own body, or allogeneic cells sourced from a donor.^2^ Stem cells, characterized by their undifferentiated nature with the capacity to develop into diverse cell lineages, stand as a potential cell source for this therapeutic approach. The strategy of implementing cell‐based therapy can be executed via direct cerebral injection of cells at the injury site.^3^ The therapeutic benefits of cell repair have been demonstrated in numerous preclinical studies, and its effectiveness is currently being evaluated in several clinical trials.^1^ However, despite the progression of cell‐based CNS reparation as a therapeutic strategy throughout the years, substantial barriers, including immune rejection, difficulty in targeting specific brain regions, and potential risks of tumorigenesis, have impeded its widespread implementation in clinical settings. Hence, as an alternate, cell‐based biomaterials like hydrogels, constituting gel‐like substances amenable to injection into the brain, offer a platform to facilitate cellular proliferation and viability.^4^ Hydrogels have demonstrated their suitability as biomaterials for brain tissue regeneration due to their unique characteristics. These materials feature three‐dimensional crosslinked polymer networks with a water content exceeding 90%. Additionally, hydrogels offer adjustable physicochemical properties, enabling them to conform to irregular brain cavities effectively. They create a favorable microenvironment conducive to the growth and proliferation of nerve cells. Furthermore, their porous internal structure imparts softness and flexibility, minimizing tissue damage during application. These hydrogels furnish a scaffold for cellular growth and afford immunoprotection to the cells. Beyond the mere replacement of damaged cells, cell‐based hydrogels also possess the capability to induce repair and regeneration of compromised brain tissue. This outcome may arise through the secretion of growth factors or other signaling molecules by the transplanted cells or by instigating the patient's endogenous cells into restoring and regenerating the afflicted tissue.^5^, ^6^

Traditionally, highly viscous noninjectable hydrogels like polyethylene glycol (PEG) and poly 2‐hydroxyethyl methacrylate hydrogels (poly‐HEMA) have been employed to treat CNS injuries and are adapted for tissue engineering purposes. These hydrogels are modified with substances like arginylglycylaspartic acid (RGD) and natural materials such as alginate, gelatin, agarose, hyaluronic acid (HA), and chitosan to enhance cell adhesion, making them effective biomaterial scaffolds for treating brain lesions. However, their physical properties limit their application, with systemic or direct brain administration causing cell loss or dispersion. Attempts to scale up vascular transport during implantation can worsen cerebral ischemia due to blood flow obstruction during open surgery or incisions. Moreover, implanted biomaterials often trigger neuro‐immune reactions, disrupting the BBB and causing inflammation with plasma and peripheral blood cells entering the ventricle. In the context of CNS injury, injectable biomaterials may be used to repair or replace damaged tissue or to deliver drugs or other therapeutic agents to specific locations within the brain.^7^ Nanoparticles (NPs) and microparticles are also potent injectable biomaterials used for brain injury repair. These particles have advantageous properties such as smaller size, shape, and structure.^8^ It is easier to introduce them into biomaterial matrix containing stem cells, which offers a beneficial mechanism for continuous and smooth delivery of medication.

Injectable hydrogels as potential biomaterials offer several potential advantages for the treatment of CNS injury. They can be designed to release drugs or other agents over a controlled period of time, allowing for sustained therapeutic effects.^9^ They can also be designed to be biocompatible and biodegradable since they are not harmful to the body and can be safely broken down and eliminated after use. In addition, injectable biomaterials may be able to cross the BBB, the protective layer of cells surrounding the brain.^10^ Overall, injectable biomaterials hold great promise as a means of treating CNS injury and improving the lives of individuals affected by these conditions. On this basis, this comprehensive review is drafted to provide an overview of different CNS conditions that require cell reparation. It also explores the potential biomaterial methodologies that can enhance the efficacy of treating brain injuries. Moreover, this narrative review covers various aspects of the topic, including the current state of research, available interventions, challenges, and advancements in the field.

## 2 METHODOLOGY

All relevant information concerning injectable biomaterials for CNS injury was collected from published literature in the English language. Published preclinical and clinical study results were reported in the current study. We conducted a comprehensive literature search from 2001 to 2023 in major scientific databases, including PubMed, Scopus, Science Direct, Google Scholar, and Springer Link. Among the search terms and Boolean operators used were “Injectable Biomaterials” AND “Neurodegenerative diseases; Brain injury; Brain tumor; Nerve injury”; “Injectable biomaterials” AND “Cell based therapy”; “Injectable biomaterials” AND “nanoparticles”; “Injectable biomaterials” AND “microparticles”; “Injectable biomaterials” AND “Hydrogels”, without any word limits. A total of 1535 documents were retrieved from the initial database searches. These documents underwent a rigorous screening process. Two independent reviewers initially screened titles and abstracts for relevance based on predefined inclusion and exclusion criteria. Inclusion criteria include the availability of English‐language data, strategies for repairing brain injury, diseases amenable to cell‐based therapies, along with some applications of different hydrogels for CNS injury. As well, books and abstracts that met the inclusion criteria were included in the review. Articles with insufficient or ambiguous data as well as articles written in languages other than English were excluded from consideration. After excluding such literature with insufficient data or published in a foreign language, 130 studies were finally included in the study for data extraction and synthesis. Notably, the selected 130 studies provided a comprehensive basis for synthesizing the current state of knowledge regarding the use of injectable hydrogels in CNS injury contexts. This methodology allowed us to encompass a wide range of research, ensuring the relevance and quality of the included studies in our narrative review.

## 3 CELL SOURCES

Various cell types can be employed to rejuvenate damaged CNS tissue. Table 1 outlines the remarkable benefits and extensive application of diverse cell types for repairing CNS injuries.

**Table 1:** Diverse stem cell types for neural regeneration: advantages and its limitations.

Cell type	Advantages	Limitations	References
ESCs	It is capable of differentiating into any type of cell.	There are ethical concerns and the possibility of tumor formation.	[11, 12]
iPSCs	It is capable of differentiating into any type of cell.	Risk of tumor formation.	[13]
NSCs	It is capable of differentiating into neural cells, and there is less chance of tumor formation.	The availability of this cell type is limited, and it may not integrate well with the host tissue.	[14]
Olfactory ensheathing cells	Promote nerve regeneration	The availability of this cell type is limited, and it may not integrate well with the host tissue.	[15]
Schwann cells	Promote nerve regeneration	The availability of this cell type is limited, and it may not integrate well with the host tissue.	[16, 17]

Abbreviations: ESCs, embryonic stem cells; iPSCs, induced pluripotent stem cells; NSCs, neural stem cells.

## 4 DISEASES AMENABLE TO CELL‐BASED THERAPIES

### 4.1 Spinal cord injury (SCI)

**Figure 1:**
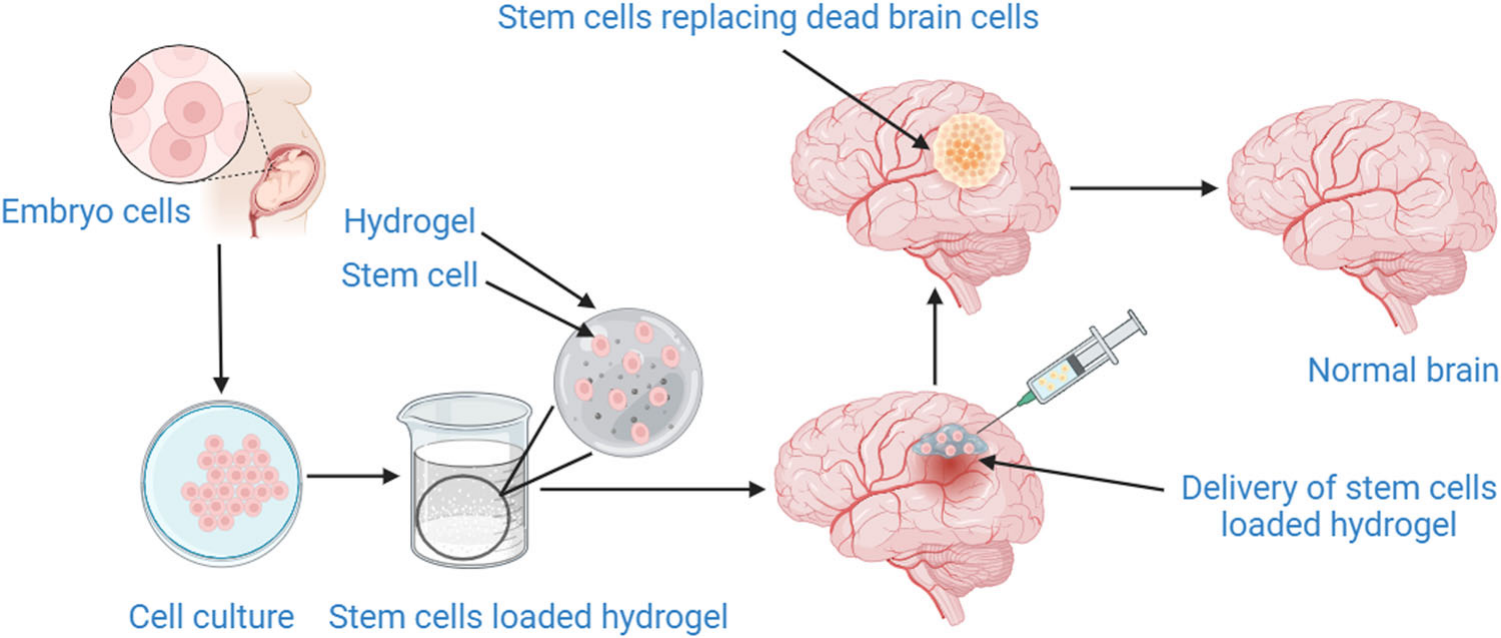
Delivery of stem cell‐loaded hydrogel to the brain. [Color figure can be viewed at wileyonlinelibrary.com]

These injuries can result in paralysis and loss of sensation due to damage to the spinal cord. Cell‐based therapies, such as the transplantation of neural stem cells, have been shown to promote nerve regrowth and improve functional recovery in animal studies. Several methods of cell transplantation are being explored as therapeutic strategies for SCI, including modulating the host immune response, enhancing axonal plasticity, providing neurotrophic support, and replacing damaged cells.^18^ It has been demonstrated that neural progenitor cells are effective at improving motor function and forming synaptic connections between grafted neurons and host neurons when transplanted into rodent and nonhuman primate models of severe SCI. In the case of SCI, numerous stem cells, including neural stem cells (NSCs), embryonic stem cells (ESCs), Wharton jelly stem cells, adipose‐derived mesenchymal stem cells (ADMSCs), umbilical cord blood stem cells (UCB‐SCs), bone marrow mesenchymal stem cells (BMSCs), umbilical cord‐derived mesenchymal stem cells (UC‐MSCs), and induced pluripotent stem cells (iPSCs), have been shown to be effective in the repair of SCI. Constructed NSCs‐derived neural network tissue with strong survival within an NT‐3/fibroin‐coated gelatin sponge (NF‐GS) scaffold, modified by tropomyosin receptor kinase C (TrkC). After receiving tissue from neural networks produced from NSCs that had been transformed by TrkC, SCI‐affected rats' recovery of motor function was noticeably enhanced. Together, the findings revealed that a useful method for researching and creating SCI treatments involves transplanting the neural network tissue created in the three‐dimensional (3D) bioactive scaffold.^19^, ^20^ Moreover, the synergistic application involves the coalescence of induced pluripotent stem cell (iPSC)‐derived neural stem cells (iNSCs) with a 3D construct of gelatin methacrylate (GelMA) hydrogel, aimed at fostering regenerative processes subsequent to SCI. A mouse spinal cord transection model was created to evaluate the GelMA hydrogels' impact on in vivo neuronal regeneration. Implants made of GelMA/iNSC greatly aided functional recovery. Additional histological examination revealed that the GelMA/iNSC group had much smaller cavity regions and less collagen was deposited. Additionally, the combination of GelMA and iNSC transplantation reduced inflammation by lowering active macrophages/microglia (CD68‐positive cells). Furthermore, the implantation of GelMA/iNSC showcased noteworthy therapeutic outcomes by forestalling the formation of glial scars and GFAP‐positive cells, concomitantly fostering the regeneration of axons. The utilization of this tridimensional hydrogel laden with stem cells stands undoubtedly as a prospective therapeutic modality for SCI reparation.^21^ Notably, rats afflicted with SCI and subjected to a dual intervention involving edaravone injection alongside Schwann cell transplantation exhibited enhanced neurological functionality coupled with the regrowth of nerve fibers.^22^ A typical representation of the delivery of stem cells into the brain is represented in Figure 1.

### 4.2 Traumatic brain injury (TBI)

TBI is still a leading cause of mortality and disability, affecting an estimated 10 million individuals each year. TBI is a disease that destroys normal brain function and produces major physical, cognitive, and emotional problems. TBI pathophysiology consists mostly of a breach of the BBB, widespread neuro‐inflammation, diffuse axonal damage, and neurodegenerative abnormalities.^23^ Preclinical research has demonstrated the potential therapeutic usefulness of stem cell therapy. In preclinical research, neuroprotective and regenerative capabilities of stem cells have been proposed as the mechanism of action.^24^ In recent years, research has discovered that a number of stem cells, including MSCs, NSCs, multipotent adult progenitor cells (MAPCs), and EPCs, can heal neurological damage following TBI.^25^ STEMTRA, a recently completed phase 2 clinical trial (NCT02416492) that observed the potential effects of intracranial administration of bone marrow‐derived cells on patients with chronic motor deficit from TBI, discovered that significantly more patients receiving cell treatment showed enhanced motor status at 6 months in comparison to the patients receiving a control sham.^10^

### 4.3 Strokes

Strokes are the third most common cause of disability and the second most common cause of mortality worldwide. Stroke, being the major cause of depression and dementia, causes brain cells to die suddenly due to lack of oxygen flow to the brain.^26^ Merely two treatments possess approval from the Food and Drug Administration (FDA) for stroke, namely tissue plasminogen activator and thrombectomy. Remarkably, despite being the second leading cause of both mortality and enduring incapacitation on a global scale, the available therapeutic options remain limited. As a multimodal therapy with the potential to produce neuroprotective and regenerative growth factors as well as potentially acting as cell replacement for lost and injured brain cell types, NSCs have attracted a lot of attention.^27^ NSCs‐based therapy may be significant among cell types due to its capacity for neuronal differentiation, as well as its capacity to promote endogenous neurogenesis, angiogenesis, and the control of the neuro‐inflammatory system.^28^, ^29^ Although cell‐based therapy may be an effective stroke treatment, there are still numerous obstacles to overcome, such as poor transplanted cell yields and the heterogeneity of the disease and injured tissue.

### 4.4 Neurodegenerative diseases

#### 4.4.1 Parkinson's disease (PD)

PD is an incurable condition that causes the dopaminergic neurons of the substantia nigra pars compacta to degenerate locally. Many of the PD treatments available today can only treat the symptoms, not the underlying neurodegeneration that causes PD. MSCs, ESCs, NSCs, and iPSCs‐based cell treatment have been the subject of numerous animal research.^30^, ^31^ The efficacy of cell replacement therapy for PD is widely supported by these preclinical investigations, providing a viable substitute for pharmaceutical therapies. Additionally, cell repair has been used in numerous clinical investigations for PD. For instance, the European research group TRANSEURO was founded in 2010 (NCT01898390) to examine the therapeutic effectiveness of utilizing transplants of the fetal ventral mesencephalon to replace cells in PD.^10^, ^32^ Using iPSCs, recent developments in the fields of cellular reprogramming and personalized medicine now enable the development of cell therapies that were previously unreachable and the patient‐specific modeling of PD using iPSCs.^33^ iPSCs can be specifically differentiated to become dopaminergic neurons, which are prone to neurodegeneration naturally. The first striking human trial for iPSC transplantation in PD started in Japan in 2018. One of the strongest possibilities for slowing or perhaps reversing the progression of PD at the moment is this type of cell treatment, which has demonstrated encouraging outcomes in other model organisms.^34^

#### 4.4.2 Alzheimer's disease (AD)

AD is the most prevalent neurodegenerative disease characterized by amyloid (Aβ) plaques that eventually form in degenerating neurons of the aged brain. These protein plaques are mostly made up of Aβ fibrils and neurofibrillary tangles (NFTs) of phosphorylated tau protein. Despite the development of several cholinesterase inhibitors, *N*‐methyl‐d‐aspartate (NMDA) receptor antagonists, and monoclonal antibodies to prevent neurodegeneration, trigger neural regeneration, or clear up Aβ deposits, none of the treatments are helpful in alleviating the cognitive and memory dysfunctions of AD patients. As a result, stem cell therapy represents a potent treatment option for AD.^35^, ^36^ AD stem cell sources include NSCs, ESCs, and MSCs derived from bone marrow, umbilical cord, and umbilical cord blood. Patient‐specific iPSCs in particular are presented as a future potential and a challenge for the treatment of AD.^37^, ^38^ As a replacement or regeneration method, stem cell‐based treatment provides new promise for AD treatment. MSCs are multipotent stem cells with immunomodulatory capabilities and excellent biosafety, as well as the potential to synthesize neurotrophic and proangiogenic molecules, making them effective for neuro‐regeneration.^39^ In the instance of AD, MSCs‐based therapies have demonstrated the ability to heal damaged brain tissue while also slowing disease progression. Although the exact treatment mechanism effect of stem cell transplantation is unknown, MSCs therapy has demonstrated to be an alternative therapeutic option for neurodegenerative illnesses such as AD. Several clinical trials are being conducted to evaluate the utility of MSC infusions in patients with AD.^40^ For example, the Korean business Medipost conducted a phase 2 clinical trial (NCT02054208) in patients with AD to evaluate intra‐ventricular delivery of a product named NEUROSTEM®, (UCB‐MSCs). Another product, Astro Stem, employs ADMSCs and will shortly be evaluated in patients with AD in a phase 2 clinical trial (NCT04482413).^10^ Similarly, NSCs transplantation, which targets both neuron networks and pathogenic proteins, has a positive effect on behavior and the microenvironment. NSCs stimulate endogenous synaptogenesis, which improves cognition. However, it is challenging to correctly characterize NSCs' positive effects in AD.^41^

#### 4.4.3 Multiple sclerosis (MS)

Exceeding a global count of 2 million individuals, MS affects a substantial populace and manifests as a persistent inflammatory disorder of the CNS. The intricate nature of MS arises from the interplay between genetic propensities and environmental hazards, contributing to its onset. A variation of the HLA‐DRB1 gene of the major histocompatibility complex is the key genetic determinant linked to MS susceptibility, but there are numerous other minor genetic risk factors as well.^42^, ^43^ The term “stem cell therapy” refers to a vast field that includes the therapeutic transplantation of numerous stem/progenitor cell types derived from diverse tissues into potential patients. Hematopoietic stem cells (HSCs) transplantation^44^ is presently the only method with clinical validation in this area. This method, which is frequently used to treat hematologic malignancies including multiple myeloma and leukemia, is also showing amazing results in patients with active RRMS who do not react to DMTs.^45^

## 5 BIOMATERIALS FOR BRAIN REPAIR

**Figure 2:**
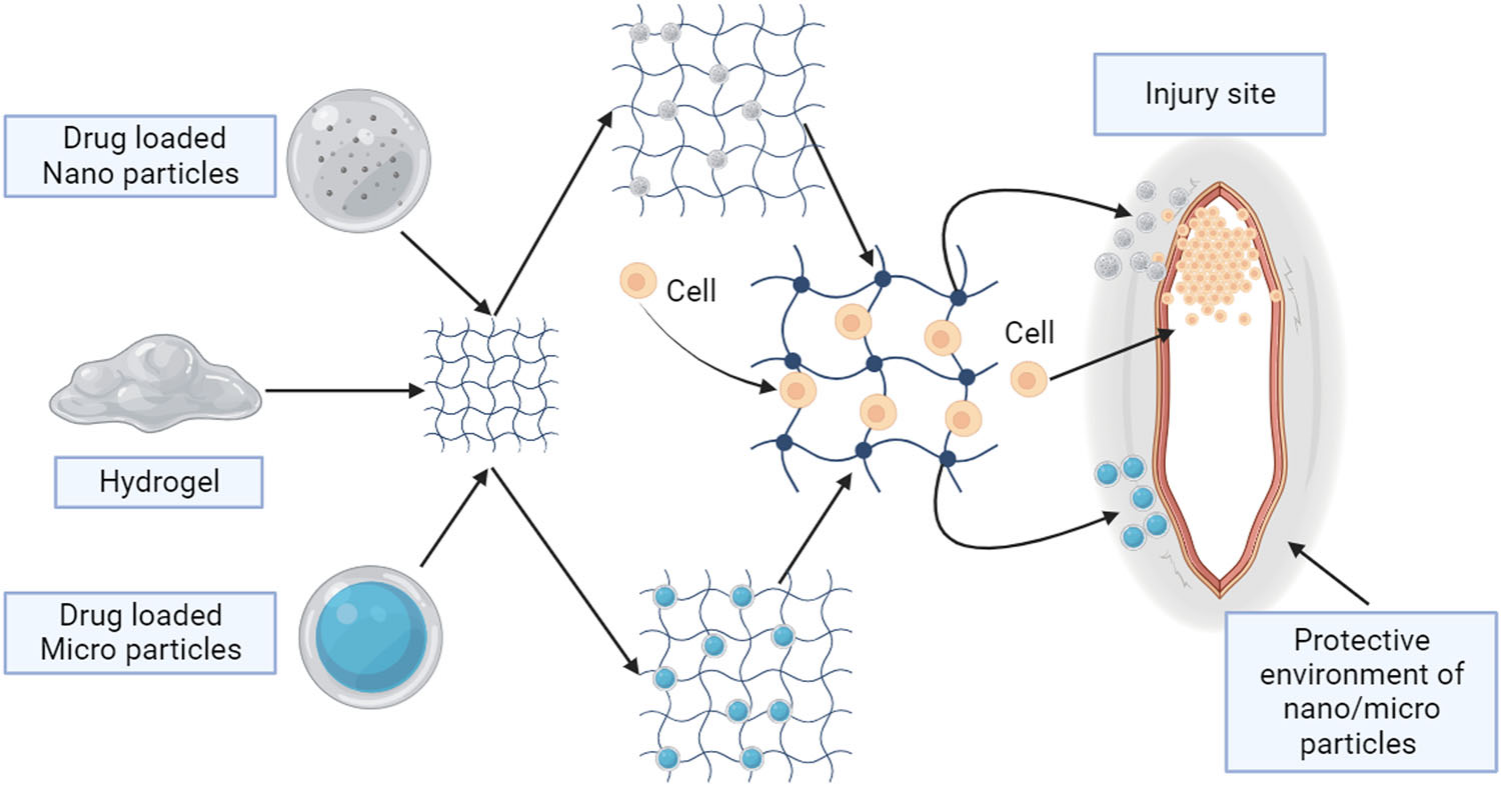
Characteristics, similarities and differences of these biomaterials in central nervous system injury. [Color figure can be viewed at wileyonlinelibrary.com]

In the context of CNS injury, injectable biomaterials serve the purpose of repairing or replacing damaged tissue. Additionally, they enable precise drug delivery to specific locations within the brain. Different biomaterials including cell‐based hydrogels, microparticles, NPs are effectively used for targeted delivery of therapeutics to the brain, which are discussed as follows. Moreover, a pictorial representation illustrating the characteristics and similarities and differences of these biomaterials is shown in Figure 2.

### 5.1 Cell‐based hydrogels

The brain is an intricate part of the CNS, responsible for integrating and regulating information in the nervous system along with the spinal cord. Injuries to the CNS due to diseases, disorders, or accidents are very critical as it hardly regenerates.^46^ The rapid movement of astrocytes towards injured sites prevents further damage to the neural network by forming glial scars around the injured sites. The brain damage became irreversible due to inadequate regeneration of neurons and axons and the development of glial scars at the lesion.^47^ Facilitating the recovery of neuron and axon regeneration by NSCs transplantation along with inhibitory antibodies is quite a hurdle, preventing its pervasive clinical practice.^48^ However, due to inadequate physical support to the lesion as well as microenvironment cherishing the cell growth makes neural cell transplantation scant for substantial brain injury. A variety of biomaterials with physicochemical properties that can be altered in response to the environment, such as malleable biodegradability, tissue‐like mechanical properties with structural resemblance to extracellular matrix (ECM), pertinent bioactivity, and cytocompatibility, have emerged into focus for the purpose of providing a microenvironment for cell growth and tissue regeneration of soft tissue, bone, and the peripheral nervous system as well as the CNS.^49^, ^50^ The ideal material for regenerating brain tissue has proven to be hydrogels, as they contain 3‐dimensional crosslinked polymer networks with a water content above 90%. Moreover, hydrogels possess adjustable physicochemical properties for filling the irregular cavity in the brain. It can also provide a favorable microenvironment for nerve cell growth and proliferation; apart from that, the porous internal structure provides its softness and suppleness, leading to the minimization of tissue damage.^10^ Traditionally, non‐injectable, very viscous hydrogels have been used to treat CNS injuries. In tissue engineering, such hydrogels are frequently converted into external wound dressings or implantable biomaterial scaffolds.^51^ Noninjectable hydrogels with mechanical qualities like those of brain tissue are commonly utilized in the regeneration of brain tissues, which includes PEG and poly‐HEMA. After being modified with the RGD sequence and natural substances including alginate, gelatin, agarose, hyaluronic acid (HA), and chitosan, the capacity of hydrogels to adhere to cells dramatically increases, enhancing their effectiveness as biomaterial scaffolds for treating brain lesions.^52^ However, the physical characteristics of non‐injectable hydrogels limit the range of applications, when administered systemically or directly into the brain, most transplanted cells perish or spread to the peripheral organs. Furthermore, scaling up vascular transport of stem/progenitor cells might aggravate cerebral ischemia by obstructing blood flow to the brain, since implantation is only possible through an open surgery or requires a substantial incision to be made.^53^ Also, the implanted biomaterials frequently cause neuro‐immune reactions, causing the BBB to be broken and plasma and peripheral blood cells to enter the ventricle. Inflammation and neuro‐immune reactions can be brought on by an influx of fluid and peripheral blood cells, such as lymphocytes and macrophages.^54^ Drugs or cells can be directly delivered by injectable hydrogels to the damaged area of the brain, protecting the BBB. The CNS may be treated with drugs using injectable hydrogels, and neuronal stem cells can be transported using these hydrogels as scaffolds.^55^ As minimally invasive surgery continues to advance, the usage of injectable hydrogels adapts to the brain's environment and satisfies the evolving needs of biomaterials for smaller surgical sites. The in situ injection of hydrogels promotes gelation, which results in the clearing of cerebrospinal fluid (CSF) and restricts extensive dissemination to maintain local release of hydrogel in the lesion region, might be accelerated by revamping the physical and chemical characteristics.^56^, ^57^ With every new technological advancement, hydrogels design quickly develops, enhancing every component of cell repair (cell adhesion, biological signals) to deliver the most effective neuroprotective result to the targeted cells.

### 5.2 Nano and microparticles

Novel drug administration of liposomes, polymeric micelles, and NPs have the potentials to be powerful tools for addressing CNS injuries. NPs containing germane molecules are suitable biomolecules, and an important resource for in situ administration in the CNS. Because of their tiny size, encapsulated biomolecules containing active chemicals can traverse the BBB, improving pharmacological profile while limiting toxicity.^58^ Due to limited regenerative impact of delivering biomolecules alone in damaged tissue, nanoparticle unaided approach is not befitting for tissue regeneration in CNS. However, the potential of micro‐carriers and other microscale materials for cell‐based brain healing in the brain has been demonstrated by a variety of research.

Formulations of polymeric micro/nanoparticles for the controlled release of hydrophobic and hydrophilic biomolecular medicines have been extensively developed. The preservation of biomolecule activity and biomolecule diffusion from the delivery site to the target site are challenges in the design of micro/nanoparticle formulations for CNS regeneration. The majority of micro/nanoparticles deployed in the CNS are made of poly‐lactic‐co‐glycolic acid (PLGA), a biodegradable polymer that has been recognized for use in medicine. After being injected into tissue for CNS distribution, PLGA micro‐particles cause a modest microglial/astroglial response and can linger there for up to 4 months.^59^, ^60^ The use of nerve growth factor (NGF) or GDNF‐containing PLGA micro‐particles for the treatment of PD, AD, and retinal degeneration is widespread.^58^ The neurotrophins were released in vitro over a period of 6 weeks with a high burst release typical of PLGA micro‐particles, and this led to both tissue regeneration and functional gains. It is undesirable because surfactants can be cytotoxic that high quantities of surfactants were needed to disperse the micro‐particles for injection.^61^ Another strategy is to distribute both cells and medicinal compounds using pharmacologically active micro‐carriers. One such instance is the in vitro seeding and in vivo transplantation of fetal ventral mesencephalic transplants in the hemi‐Parkinsonian rat striatum using PLGA micro‐carriers functionalized with GDNF.^62^, ^63^ This method increased the cells’ capacity for functional function as well as for reinnervation and survival. In preclinical models of stroke, pharmacologically active micro‐carriers have also been employed to increase the reparative capability of a population of MSCs known as marrow‐isolated adult multilineage inducible (MIAMI) cells,^63^, ^64^ PD^62^ and Huntington's disease.^65^ Apart from that another approach was made earlier to embryonic rat hippocampal neurons which were effectively matured and transplanted into the adult rat hippocampus using colloidal glass microbeads.^66^

### 5.3 Hydrogel containing micro/nanoparticles

Approaches are being developed to combine micro/nanoparticles acting like carriers along with cell‐loaded hydrogels, such as the preparation of a MnO_2_ NPs dotted hydrogels involve dispersing MnO_2_ NPs in a PPFLMLLKGSTR peptide in modified hydrogels made of hyaluronic acid. MSCs can adhere to the hydrogels' surface and bridge nerve tissue with it. The MnO_2_ NPs successfully increase MSCs viability by reducing the oxidative environment. On a long‐span rat spinal cord transection model, MSCs transplantation results in a significant recovery of motor function. It also causes in vivo integration and neural differentiation of the implanted MSCs, which results in a highly effective regeneration of CNS spinal cord tissues. Therefore, by the thorough management of pathological microenvironment problems, the MnO2 NPs dotted hydrogels represents a promising technique for stem‐cell‐based therapy of CNS illnesses.^67^ Also, a significant potential has been introduced to treat pathological imbalance of zinc during epilepsy and dementia by using a hydrogel made of HA generated from skeletal muscle, ZnONPs are delivered locally to the damaged spinal cord (ZnONPs‐Gel). In this study, ZnONPs‐Gel implantation successfully restored hindlimb motor function in SCI mice by controlling the focal microenvironment and reducing reactive oxygen species and inflammation. This work created a novel method for administering ZnONPs locally to treat SCI.^68^ The hydrogels maintain the cells and particles in the treatment location while the particles provide a continuous, long‐term release of the biomolecules, such as neurotrophic factors to boost cell survival. This synergistic method offers several benefits. Numerous research on PD have demonstrated the positive effects of neuronal cell transplantation on GDNF‐rich scaffolds, which enhance survival and functional recovery.^69^ Although the primary goal of utilizing nanoparticles is often to increase cell survival, therapeutic molecules can also be utilized to improve other features, such as angiogenesis by introducing, for example, VEGF in the wounded site.^70^, ^71^

## 6 TYPES OF INJECTABLE HYDROGELS AND ITS APPLICATION IN BRAIN INJURY

Injectable hydrogels extensively used in brain injury research are classified into natural injectable hydrogels, synthetic hydrogels and self‐assembled or mixed hydrogels. Each category possesses unique attributes that make them suitable for specific applications. They differ in terms of their composition, origin, properties, and applications that are well‐discussed within this review.

### 6.1 Natural injectable hydrogels

Natural injectable hydrogels, derived from biocompatible materials like cellulose, hyaluronic acid, collagen, chitosan, and others closely mimic the native CNS extracellular matrix. Their inherent biocompatibility and biodegradability make them an attractive choice for CNS repair. These hydrogels foster neural regeneration by creating a conducive microenvironment, promoting cell adhesion, and minimizing immune responses. The most commonly used natural hydrogel materials in tissue engineering are based on the natural components of ECM, such as cellulose, hyaluronic acid, chitosan, collagen, alginate, and gelatin,^72^, ^73^ which are discussed in detail below.

#### 6.1.1 Injectable ECM

Aside from its low immunogenicity and 3D structure, native ECM hydrogels have a multitude of biomolecules, including cytokines and chemical substances that stimulate cell growth and differentiation.^49^, ^74^ ECM is generally digested with pepsin and decellularized to prepare injectable ECM. Materials for preparing ECM hydrogels fall under two categories, namely, (a) nerve tissues and (b) non‐nerve tissues.^50^, ^75^ A study by Ghuman et al.^76^ developed an injectable fluid by preparing ECM from pig bladder, exposing it to 4% ethanol, decellularizing it with 0.1% acetic acid, and lysing it with pepsin at a temperature of 21°C. The biodegradation rate of ECM hydrogels, the degree to which endogenous cells and nerve cells penetrated the materials, and the degree to which tissues were modified around the apoplexy cavity were determined by injecting different concentrations of the hydrogels into apoplexy rats' brains. The results of these studies indicate that injectable ECM hydrogels in the dosage of 4 mg/ml can result in maximum tissue regeneration in the brain. After 14 days of implantation, 80% of the ECM hydrogels degraded at a rate of 6.11 litres per day. There was a steady infiltration level of macrophages in the hydrogels that could be maintained at 700–800 cells/μL. It has been reported that the remaining hydrogels became denser with mature nerve cells after 90 days of injection which is a sign of tissue transformation and regeneration.^76^ Such hydrogels functions as a supportive scaffold when injected into damaged neural tissue, providing a conducive microenvironment for cell attachment, proliferation, and survival. This biomimetic platform promotes neuroplasticity, aids axonal regeneration by guiding nerve fibers,^74^ modulates immune responses to curb excessive inflammation, and can deliver bioactive molecules to facilitate tissue healing. Through these mechanisms, injectable ECM holds potential for ameliorating debilitating neurological conditions by promoting repair and functional recovery within the nervous system.^49^, ^50^

#### 6.1.2 Hyaluronic acid

The polysaccharide HA is highly aqueous with repeated and alternate disaccharide units, mostly located in the brain and the ECM.^77^, ^78^ The biocompatibility and biodegradability of HA makes it ideal for use as a base for the development of new biologics. Additionally, it is used for cell transplantation, or it can be used on its own as a biological scaffold.^51^ It exerts potential neuroprotective effects by modulating neuroinflammation and promoting tissue repair. Through interactions with CD44 receptors, HA regulates immune responses and dampens inflammation, thus reducing neuronal damage. Moreover, the viscoelastic properties of HA can contribute to tissue restoration by providing structural support and promoting cellular regeneration in debilitating neurological conditions.^51^, ^78^ Studies have prepared HA‐based hydrogels by crosslinking MMPase peptides with acrylate and adding HA to the main chain. It is possible to mimic brain mechanical properties by varying the cross‐linking agents.^52^ According to a study, human pluripotent stem cells injected into stroke mice stimulated differentiation of the cells, but after 1 week, the hydrogels failed to improve the survival of stem cells.^53^ In contrast, in another study, HA hydrogels containing brain‐derived neurotrophic factor (BDNF) were injected into the cranial cavities of mice within a week of stroke. The group receiving HA hydrogels combined with BDNF resulted in a significantly higher number of new neurons in contrast to the control group following 9 weeks of stroke.^54^ This suggested that HA hydrogels with BDNF significantly increased the number of new neurons in the brain.

In addition to these pros, Ho et al.^55^ reported that hyaluronic acid‐methylcellulose (HAMC) hydrogels, which are HA based physical cross‐linking hydrogels, cause severe damage to the CNS. It was reported that erythropoietin delivered via hydrogels to the cerebral cortex of stroke induced mice resulted in a significant increase in neurons, a decrease in inflammation, a reduction in stroke lumen size, and migratory neuroblast proliferation in the inferior ventricle.^55^

#### 6.1.3 Chitosan

Chitosan, a natural polymer mainly composed of d‐glucosamine and randomly linked β‐(1‐4) units of *N*‐acetyl‐d‐glucosamine, is recognized as the most abundant polysaccharide after cellulose for its biodegradability, biocompatibility, non‐allergenicity, non‐toxicity, resistance to oxidation, antibacterial and anti‐inflammatory properties.^56^ It has shown potentials in mitigating debilitating neurological conditions through several mechanisms. Firstly, chitosan's positively charged amino groups can interact with negatively charged molecules, potentially reducing the aggregation of misfolded proteins implicated in neurological conditions like AD. Secondly, its anti‐inflammatory properties may modulate neuroinflammatory responses, offering neuroprotection. Lastly, chitosan‐based drug delivery systems could enhance the targeted delivery of therapeutic agents across the BBB, facilitating treatment strategies for neurological disorders.^56^, ^58^ However, for chitosan‐based biomaterials, functionalization is necessary to address its low solubility and poor mechanical properties. For instance, adding succinic or ferulic acid to chitosan hydrogels injection and administering it to Wistar rats demonstrated adequate biocompatibility. This suggests the potential application of functionalized chitosan hydrogels to treat CNS injuries.^57^ Tseng and his colleagues in 2015 synthesized a self‐injectable chitosan‐based hydrogels that gelatinized slowly at room temperature but rapidly at body temperature (37°C). The hydrogels stimulated 38% neural regeneration in the zebrafish embryo model.^58^ Yao and colleagues, by combining chitosan, beta‐glycerophosphate, hyaluronic acid and hydroxy cellulose developed a thermal hydrogel carrying human umbilical cord neural stem cells. This biocompatibility composite thermal hydrogel with optimum gelation rate secretes BDNF resulting in neuronal proliferation, inhibiting apoptosis process, facilitating survival and recovery in rat models.^59^

#### 6.1.4 Fibrin

Fibrin, a natural enzyme‐degrading protein synthesized by simply mixing thrombin with fibrinogen and involved in blood and lymph coagulation is highly biocompatible and biodegradable in nature.^60^ Its ability to bind many proteins, cells, and ECMs makes it a flexible hydrogel.^79^ Additionally, the ratio of fibrinogen and thrombin can be adjusted to match the mechanical properties of fibrin hydrogels with those of human spinal cord tissues. Furthermore, as per study, during in vivo degradation or polymerization, the released by‐products from the hydrogel also contribute to damage repair.^79^

By the year 1998, the US‐FDA had approved fibrin tissue sealant for use in intraoperative hemostasis and wound healing.^80^ By introducing ultrasound into a silk fibroin solution, some researchers developed a silk hydrogel based on fibrin. By controlling the time and intensity of ultrasound, brain compatibility was achieved. Experimental mice injected with this silk‐based fibrin hydrogel showed cell death and inflammation in the implanted area without any sensory, motor, or cognitive deficits, indicating its biosafety in the brain.^81^ Further study has demonstrated that fibrin‐based biological scaffolds are capable of not only providing a sustaining environment for transplanted cells after a brain injury but also exerting neuronal antiapoptosis.^82^ When used homologously, this hydrogel has been found to have complete biocompatibility based on current research. Neuronal differentiation could be enhanced by precisely controlling its mechanical properties by adjusting the ratio of fibrinogen to thrombin or by adding some other stable hydrogels.^83^ In light of these characteristics, fibrin is an interesting tissue engineering candidate, however, fibrin hydrogel systems have been poorly utilized in brain research to date.

#### 6.1.5 Collagen

Collagen, a natural hydrogel synthesized by hydrolysis of gelatin, has diverse applications in medical science such as tissue engineering, drug delivery, bone reconstruction, dressing of wounds and various cell encapsulation systems. Depending upon applicability, it can be shaped from films and shields to nanoparticles, sponges and gels.^84^, ^85^ While there are 29 types of collagen, only type I and type IV collagen are abundant and most widely used.^86^ Type IV collagen plays an important role in the formation of neuromuscular junctions and the basement membrane of the BBB within the nervous system. While type I collagen contributes to development of neurons, formation of dura mater, pia mater as well as growth of axons.^87^ It has also gained immense attention for CNS repair owing to its biodegradability, biocompatibility, versatility, and nontoxicity and is considered a potential candidate for regeneration of brain tissues.^85^

Based on applicability, collagen can be shaped to micro or nanospheres or injectable scaffolds to release drugs locally to protect neurons in neurodegenerative conditions like PD or AD and for encapsulating stem and genetically modified cells for its safer delivery. Further, collagen has immense application as filling agents in filling the voids caused by injuries where it provides structural support and a suitable microenvironment for the growth of axons.^88^ Literature provides sufficient information regarding use of collagen hydrogels in brain tissue regeneration therapy. In the study by Guan and his colleagues, human bone marrow‐derived mesenchymal stem cells (hMSCs) in combination with collagen hydrogel were injected into the injury site of experimental rats.^89^ Results showed that the combination of collagen hydrogel with hMSCs is therapeutically more promising as compared to the hMSCs alone. In the presence of collagen hydrogels, the proliferation of hMSCs to specific organs was enhanced while inhibiting proliferation to nonspecific organs and supporting cell differentiation and growth. This resulted in enhanced brain metabolism and restoration of its function. In another study, rat NSCs were injected in healthy rats by suspending in collagen hydrogel solution.^90^ The stem cells displayed excellent viability in the hydrogel and could be reabsorbed after 15 days which suggests biodegradability of the collagen hydrogel.

Hoban et al. used collagen (type I) as vectors to deliver glial cell line‐derived neurotrophic factors (GDNF) to the brain via transgenic bone marine‐derived MSCs for treatment of neurodegenerative diseases. As a result of the study, hydrogel reduced both astrocyte recruitment and responses of microglial cells without affecting cell survival or GDNF secretion.^91^ These studies suggest that injectable collagen hydrogels have great potentials as a treatment for TBI and other nerve injury repair strategies. The applications and specific‐characteristics of these natural hydrogels are listed in Table 2.

**Table 2:** Versatile natural injectable hydrogels for neurodegeneration: Characteristics and applications.

Material	Characteristics	Applications	References
Injectable ECM	Brain tissue regeneration, biocompatibility	Stroke	[76]
Hyaluronic acid	Biodegradability, biocompatibility, neuronal differentiation, neurite outgrowth and promotion of ips‐NPC differentiation	Traumatic brain injury, stroke	[92, 93]
Chitosan	Biodegradability, biocompatibility, porous, antioxidant, neural differentiation, self‐healing, and anti‐inflammatory	Traumatic brain injury	[57, 94]
Fibrin	Biodegradability, biocompatibility, neuronal differentiation, neurite outgrowth porous, anti‐inflammation, and providing microenvironment for cells	Traumatic brain injury, Alzheimer's disease	[81]
Collagen	Biodegradability, biocompatibility, neuronal differentiation, neurite outgrowth porous, reducing spread of transplanted cells to other nonspecific organs	Traumatic brain injury, Parkinson's disease	[89, 90]

Abbreviations: ECM, extracellular matrix; ips‐NPC, induced pluripotent stem cell‐derived neural progenitor cells.

### 6.2 Synthetic injectable hydrogels

These hydrogels are artificially synthesized in a laboratory using chemical processes. They are composed of synthetic polymers or materials that are not naturally occurring, however it present opportunities for precise customization of properties. While modifications may be needed for optimal biocompatibility, their versatility in mechanical attributes is particularly advantageous.^95^, ^96^, ^97^ These hydrogels predominantly comprise polyacrylamide (PAM) and PEG as their base materials. In contrast to naturally occurring hydrogels, these hydrogels exhibit comparatively inferior physical characteristics and are more prone to inducing inflammatory responses following in vivo administration.^95^ They hold promise in mitigating debilitating neurological conditions through multifaceted mechanisms. These hydrogels, when injected into the affected area, can provide a supportive three‐dimensional matrix that promotes cell survival and tissue regeneration.^95^ Moreover, their tunable properties enable controlled release of therapeutic agents, such as neurotrophic factors or drugs, facilitating localized treatment and fostering neural recovery.^95^, ^97^ In an in vivo investigation conducted by Tamariz et al.,^96^ PEG‐Si, a thixotropic hydrogel containing irradiation silica nanoparticles, was intracranially administered into the striatum of rats, while a sterile saline solution was injected into the corresponding cerebral hemisphere as a control. After a duration of 30 days, notable substantiation and astrocolloid reactions were observed specifically in the hemisphere where the polymer hydrogel was introduced. In 2008, Bjugstad et al.,^97^ conducted a study involving the implantation of PEG‐based hydrogels into the striatum and frontal cortex regions of primates. The hydrogel formation involved the photocrosslinking of a PLA‐B‐PEG‐B‐PLA triblock polymer using a methacrylate group. In one hemisphere of the grivet brain, the hydrogels were injected, while the contralateral hemisphere received a needle injection without the hydrogels, serving as the sham‐implantation group. A third grivet received bilateral injections of PEG‐GDNF. The complete degradation of all hydrogels was observed after a 4‐month period. A PEG hydrogel concentration of 13% w/v induced minimal infiltration of astrocytes and microglia, which was comparable to that observed in the sham‐implantation group. Conversely, a marginal increment in the glial response was observed when utilizing 20% w/v PEG hydrogels incorporated with GDNF, thereby suggesting that PEG‐based hydrogels persist in their capacity as a viable drug delivery system with promising prospects, even subsequent to modifications.^97^

### 6.3 Self‐assembled hydrogels/mixed hydrogels

Self‐assembled hydrogels represent a three‐dimensional framework composed of polymeric substances that undergo self‐assembly through either physical or chemical crosslinking mechanisms. They may incorporate both natural and synthetic components depending on the design. These materials exhibit exceptional attributes such as remarkable biocompatibility, biodegradability, and responsiveness to physiological stimuli, thereby rendering them highly suitable for applications in tissue culture, drug delivery systems, and the fabrication of implanted sensors for human use. Furthermore, the versatile bonding interactions between the polymeric chains and water molecules, along with their capacity for chelating metal ions, expand their potential applications in fields such as photovoltaics and optics.^98^ The biocompatibility of self‐assembled hydrogels is attributed to some extent to their composition, which generally includes biological or bio‐inspired molecules such as proteins, peptides, amino acids, carbohydrates, therapeutic agents, and oligonucleotides.^99^ Such hydrogels are generated through the spontaneous self‐assembly of monomeric constituents, leading to the formation of polymer‐like fibrils through noncovalent interactions. The entanglement of these fibrils further results in the formation of an extensive network, inducing gelation of the surrounding aqueous solvent. Self‐assembled hydrogels have demonstrated distinct advantages over covalently bonded hydrogels, making them desirable for applications in the biomedical domain.^99^ The versatility and biomimetic nature of self‐assembling peptides (SAPs) render them an optimal choice for addressing the intricate and sensitive environment associated with neural tissue damage. In addition to the finely tailored properties of these hydrogels, which strive to emulate healthy physiological tissue, the use of a minimally invasive delivery approach becomes crucial to mitigate off‐target effects and complications arising from surgical interventions. SAPs, belonging to the class of injectable hydrogels, are characterized by short, repetitive amino acid units and modified polar and nonpolar residues, enabling them to form a double‐β‐sheet structure upon dissolution in water.^100^ This structural arrangement facilitates sol‐gel transition without the need for toxic cross‐linking agents or chemical additives, thereby exhibiting inherent biocompatibility. SAPs provide a favorable combination of in situ polymerization, adaptability for biofunctionalization, the capability to finely adjust physicochemical characteristics, and remarkable compatibility with living cells.^101^ Guo et al. conducted an experiment wherein an ionic complementary hydrogel, namely RADA16, was implanted into the lesion cavities of rats with surgically induced TBI. The objective was to evaluate its potential for reconstructing the damaged cortex. Remarkably, RADA16 exhibited successful integration with the host tissue and resulted in a significant reduction in the size of the lesion cavity after a six‐week period. Furthermore, researchers observed a notable decrease in pathological glial hyperplasia and an attenuated inflammatory response.^102^ In another study, SAP hydrogels demonstrated the capability to deliver exogenous stem cells into the brain. To achieve this, researchers modified RADA16 by attaching an adhesion motif called IKVAV, derived from laminin, and encapsulated NSCs within the modified hydrogels. Subsequently, this combination was transplanted into rats with neocortical damage caused by a perforated biopsy. After 6 weeks, the encapsulated NSCs proliferated and differentiated into neurons, as evidenced by an increase in the expression of mature neuronal markers such as β‐tubulin and microtubule‐associated protein 2 (MAP2), compared to the use of cell therapy alone. Moreover, there was an observed increase in the levels of Synapsin‐1, a potential marker of synaptic formation.^103^ Additionally, several experiments have demonstrated the ability of SAPs to transport drugs, biological agents, and other therapeutic substances into the damaged brain cavity, thereby improving treatment efficacy while maintaining favorable biocompatibility.^104^, ^105^

## 7 IDEAL PROPERTIES OF INJECTABLE BIOMATERIALS

Injectable hydrogels intended for CNS regeneration must exhibit biocompatibility, non‐immunogenicity, and a lack of detrimental effects on human cells and tissues.^106^ Furthermore, these hydrogels should promote axonal growth and elongation while gradually degrading over time, eliminating the need for additional surgical procedures to remove the scaffold.^107^, ^108^ The mechanical characteristics, encompassing stiffness and surface topography of the scaffolds, are pivotal factors in their biomaterial design. Studies have revealed that even small variations in mechanical and topographical features can profoundly influence cell fate, attachment, polarity, proliferation, migration, differentiation, and survival.^109^ In the context of neural tissue engineering, scaffolds must be able to withstand the stresses exerted by the surrounding tissues.^108^ Thus, before injection, the hydrogels should possess a lower elastic modulus than their storage modulus, while after injection, they should transit to a state where the storage modulus exceeds the elastic modulus, resulting in the formation of a soft and cohesive solid that can withstand the pressure from the surrounding tissues.^110^

The swelling ratio and degradation rate of biomaterials are influenced by various factors, including the type of polymer, chemical and mechanical properties of the biomaterial, and the presence of enzymes at the injury site. For effective CNS regeneration, hydrogels should degrade slowly and exhibit minimal swelling. The degradation rate of the biomaterial should strike a balance between early degradation, which could impact glial scarring, cause additional inflammation, and inhibit axon elongation, and late degradation, which could facilitate new tissue generation and prevent long‐distance axonal regeneration.^110^ Excessive hydrogel degradation before neural regeneration can result in swelling and a loss of mechanical injury.^111^, ^112^ Additionally, the porosity of scaffolds, as well as the diameter and interconnectivity of pores, are crucial for proper nutrient and biological molecular diffusion, waste elimination, cell attachment, and tissue growth.^113^ High swelling ratios can lead to hydrogel deformation, occlusion of the defect, and inhibition of neurite development.^114^ The incorporation of hydrophilic or electrostatically charged moieties in hydrogels can increase swelling, but it may also enhance network permeability and significantly affect hydrogel biomechanics. In the context of subdural injection of hydrogels after spinal cord or traumatic brain injuries, maximal swelling can aid in the removal of excess fluid from the parenchyma, thus preventing further inquiry.^115^ It is worth noting that pre‐swelling of micro‐particle‐based hydrogels^116^ and shape‐memory hydrogels before in vivo delivery may cause tissue damage similar to in situ swelling pressures. Moreover, increasing macromere content or crosslinking density can reduce water uptake of hydrogels, but this may compromise mechanical strength and permeability, leading to potential issues and adverse consequences.^117^

Another critical characteristic of injectable hydrogels is their solidification conditions, which should minimize tissue and cell damage, allowing solidification to occur under mild circumstances.^117^ The initial velocity of the hydrogel and various environmental parameters, including crosslinker, pH, light, and temperature that influence solidification are of great importance. These parameters must be biocompatible and enable the hydrogel to undergo gelation within seconds to minutes of injection.^117^ The gelation time of injectable hydrogels is a crucial consideration. Adequate gelation time is essential to mitigate the risk of injection needle occlusion and facilitate the interaction between the spared spinal cord tissue and the hydrogel solution before gelation. Further research is warranted to establish the optimal gelation times for diverse types of injectable hydrogels.^118^

## 8 CHALLENGES FOR DESIGNING BIOMATERIALS

Designing biomaterials for brain repair presents a range of challenges, as elucidated in recent scientific literature. Among these challenges, achieving optimal bioavailability and nonimmunogenicity of the employed biomaterials stands out prominently. Studies have emphasized the importance of selecting materials that do not elicit harmful immune response and are well‐tolerated by brain tissue.^119^, ^120^

Another challenge is promoting appropriate cellular interactions and tissue integration. Recent research has focused on developing biomaterials that can stimulate axonal growth, guide neuronal migration, and support the formation of functional neural networks.^121^, ^122^ Furthermore, achieving controlled degradation rates and avoiding excessive swelling is crucial for biomaterials intended for brain repair. Recent studies have investigated strategies to optimize the degradation kinetics of biomaterials to match the regenerative timeline of brain tissue and prevent adverse reactions such as glial scarring.^123^, ^124^

In addition, the mechanical properties of biomaterials play a crucial role in their design. Recent literature has emphasized the need to tailor the stiffness, elasticity, and viscoelastic properties of biomaterials to mimic the mechanical properties of native brain tissue, providing appropriate mechanical support and minimizing tissue damage.^125^, ^126^, ^127^

Moreover, the ability to deliver therapeutic agents, such as growth factors or drugs, within the biomaterial matrix is an ongoing challenge. Recent studies have explored various approaches, including the incorporation of drug‐release particles or the functionalization of biomaterials with bioactive molecules, to enable controlled and localized delivery of therapeutic factors.^128^, ^129^

Overall, recent scientific literature underscores the challenges associated with designing biomaterials for brain repair, highlighting the need for further research and innovation in this field to develop effective strategies for neural regeneration and functional recovery.

## 9 CONCLUSIONS

The intricate nature of the brain poses significant challenges in the development of effective treatment strategies. The failure to achieve successful drug treatments for neurological diseases has led to the emergence of alternative approaches, like cell‐based therapies, for brain healing and repair. The advent of biomaterials has profoundly revolutionized the potential of brain cell repair. The ability to design biomaterials‐based devices for localized delivery of cells and biomolecules in a minimally invasive manner has opened up intriguing possibilities for targeting neuroprotection and neuroregeneration, overcoming the limitations associated with conventional drug delivery methods for brain therapeutics. It is evident that we are entering an era of specialized and rational biomaterial design to address CNS injuries, with exhaustive investigations conducted for each specific condition to optimize the development of biomaterial‐based therapies. Traditional implanted hydrogels are no longer adequate for meeting the requirements of contemporary treatments. Injectable hydrogels have the ability to undergo sol–gel transition in vitro and in vivo. Precise injection of hydrogels, along with the necessary cells and drugs, into the lesion site using a fine needle, followed by in vivo gelation at an appropriate rate within the lesion cavity, holds the potential to minimize the cavity, promote nerve tissue regeneration, and reduce inflammation. However, the unstable and imprecise introduction of hydrogels into the body poses risks due to the demanding technical requirements associated with brain injection and the lack of reported procedures. Moreover, the understanding of the mechanism underlying nerve tissue growth stimulation remains insufficient. Therefore, in addition to encouraging continued research on the structure and properties of hydrogels, further investigations should prioritize elucidating the mechanism governing tissue responses to enhance functional recovery after hydrogel implantation. Ongoing research endeavors provide hope for the design of highly efficient injectable hydrogels for repairing brain damage.

## AUTHOR CONTRIBUTIONS

Santa Sarma and Dhruva J. Deka contributed to writing original draft. Damiki Laloo and Prakash Rajak contributed to supervision, formal analysis, and review. Dipankar Saha and Trishna Das contributed to formal analysis and validation. Purbajit Chetia contributed to resources. Alakesh Bharali and Bhargab Deka contributed to writing original draft, supervision, proof checking, revision, and editing.

## CONFLICT OF INTEREST STATEMENT

The authors declare no conflict of interest.

## ETHICS STATEMENT

Not applicable.

## Data Availability

Data sharing is not applicable to this article as no data sets were generated or analyzed during the current study.
